# Novel 1, 3-N, O-Spiroheterocyclic compounds inhibit heparanase activity and enhance nedaplatin-induced cytotoxicity in cervical cancer cells

**DOI:** 10.18632/oncotarget.8959

**Published:** 2016-04-23

**Authors:** Yanan Song, Bin Hu, Hongjie Qu, Lu Wang, Yunxiao Zhang, Jinchao Tao, Jinquan Cui

**Affiliations:** ^1^ The Department of Obstetrics and Gynecology, the Second Affiliated Hospital, Zhengzhou University, Zhengzhou, 450014, China; ^2^ The College of Chemistry and Molecular Engineering, Zhengzhou University, Zhengzhou, 450052, China

**Keywords:** cervical carcinoma, heparanase, inhibitors, 1,3-N, O-spiroheterocyclic

## Abstract

Heparanase (HPA) is an enzyme that plays an important role in cancer metastasis and angiogenesis and is a potential target for molecular treatment of tumors. We previously found that abnormally high HPA expression in cervical cancer tissues is associated with poor survival and increased lymph node metastasis. The present study was conducted to assess the utility of inhibiting HPA enzyme activity in cervical cancer treatment. Two series of 13 novel HPA inhibitors were synthesized and optimized. All tested inhibitors reduced HPA enzyme activity (IC_50_ values ranged from 4.47 μM to 47.19 μM) and inhibited the growth of HeLa cells (IC_50_ values ranged from 48.16 μM to 96.64 μM). The No. 16 inhibitor inhibited the migration and growth of HeLa and Siha cells in a dose- and time-dependent manner, and increased cell apoptosis and cell cycle G_0_/G_1_ and G_2_/M phase arrest, while decreasing the S phase cell population. More importantly, No. 16 sensitized cervical cancer cells to low concentrations of nedaplatin, decreased HPA, c-Myc and h-TERT levels, and increased p53 levels in HeLa and Siha cells. These results suggest that this HPA inhibitor reduced proliferation and HPA expression in cervical cancer cells by restoring p53 activity and downregulating h-TERT and c-Myc expression.

## INTRODUCTION

Currently, chemotherapy is the main treatment for relieving symptoms and reducing tumor load in cervical cancer patients with advanced, recurrent, and persistent diseases; however, mono-chemotherapy treatments do not increase the total patient survival rate [1]. Although cisplatin combined with paclitaxel extended survival time by 3 months [2], there are no second- or third-line drugs available when this combined treatment fails. The development of new agents, including targeted molecular therapies, with different mechanisms of action is crucial for improving the treatment of cervical cancer.

Clinical trials have been conducted using drugs that target several different biological agents, including vascular endothelial growth factor and its receptors, histone deacetylase, matrix metalloproteinase, cell cycle checkpoint molecules, notch, and mammal target of rapamycin [3, 4]. The GOG240 clinical study was the first to show that targeted drugs can be effective for treating patients with advanced, recurrent, and persistent cervical cancer [5]. Although anti-angiogenic agents are effective in treating some cervical cancer patients, changes in complementary biological pathways can make cancer cells resistant to these treatments. Regardless, previous research has demonstrated the utility of targeted anti-angiogenic therapies in treating cervical cancer. Here, we investigated heparanase (HPA), which plays an important role in cervical cancer metastasis and angiogenesis, as a molecular target for cervical cancer treatment.

HPA is an endoglucuronidase responsible for the degradation of heparan chains (HS). It can regulate the structure and function of heparin sulfate proteoglycan (HSPG), and thereby alter the structure of the extracellular matrix. HPA also releases biologically active fragments of polysaccharides and various cytokines and growth factors that can combine with HS. As a multifunctional protein, HPA promotes the expression of many genes (such as VEGF, TF, MMP-9, HGF, PANKL, and TNF-α) and alters the activity of signal transduction pathways (AKt, Src, Erk, EGFR, phosphorylation of insulin receptor) through enzyme activation or deactivation, and may thus be involved in multiple human pathological processes [6, 7]. HPA expression is up-regulated in almost all human malignant tumors. Studies both *in vivo* and *in vitro* have shown that the overexpression of HPA can stimulate tumor growth. Additionally, knocking down HPA expression can inhibit the growth of transplanted tumors and decrease the density of tumor vessels and lymphatic ducts. Importantly, HPA is the only human enzyme with heparanase activity, and no other molecule can compensate if it is inactivated. Due to very low expression of HPA in normal tissues, blocking HPA function does not cause serious side effects in normal subjects. Small-molecule inhibitors, a neutralizing monoclonal antibody, and modified heparin have been used to inhibit HPA and effectively treat cervical cancer in preclinical studies. Additionally, some HS analogue inhibitors have been used in clinical trials [8].

Cervical cancer is closely associated with the overexpression of HPA. In 2003, Shinyo *et al*. [9] showed for the first time that HPA mRNA expression is increased in advanced cervical cancer, and that HPA expression is higher in patients with vessel and lymphatic duct involvement. They also found that tumor micro-vessel density is related to the expression of HPA, and HPA-positive patients had shorter tumor-free and overall survival. Multiple factor analysis showed that HPA expression is an independent prognostic factor. Immunohistochemistry also confirmed that HPA levels are associated with cervical cancer tumor size and disease stage. High HPA expression *in vitro* can inhibit apoptosis in cervical cancer cells and promote cell proliferation and growth [10]. Our previous research showed that 85% of cervical cancer primary tumors and associated lymph node metastases express HPA, and patients with HPA-positive lymph nodes had shorter median survival times. COX model multi-factor analysis showed that both lymph node metastasis and HPA expression are independent risk factors (to be published in Oncology Letters). High expression of HPA in cervical cancer tissues can degrade side chains of HS-GAG associated with perlecan on the basement membrane surface [11, 12], which causes the spread of cervical cancer cells to lymphatic ducts. Dynamic contrast-enhanced MRI has confirmed that HPA can cause early vascular changes in the primary tumor and lymph node metastases [13]. The above studies strongly support the utility of targeting HPA for cervical cancer therapy.

Basappa *et al*. [14] imitated the sugar residues of the monosaccharide skeleton structure of HS to synthesize a novel pyranoside analogue, DMBO (2-(2,6-difluoropheny)-5-(4-methoxyphenyl)-1-oxygen-3-nitrogen [5,5]decyl hydride). In addition to inhibiting the HPA enzyme, DMBO also can bind to various growth factors closely associated with HPA and suppress tumor cell growth, metastasis, and angiogenesis both *in vivo* and *in vitro*. Here, we synthesized novel molecular inhibitors of HPA through structural optimization of DMBO. These new compounds effectively inhibited HPA activity (IC_50_ less than 10 μM for the strongest inhibitor). All of the compounds reduced HeLa cell growth *in vitro*. In addition, compound number 16 reduced the expression of HPA in both HeLa and Siha cells. Our study provides support for the use of drugs that target HPA as molecular therapies for cervical cancer.

## RESULTS

### Concentration inhibition curves of the HPA inhibitors and IC_50_ values

**Figure 1:**
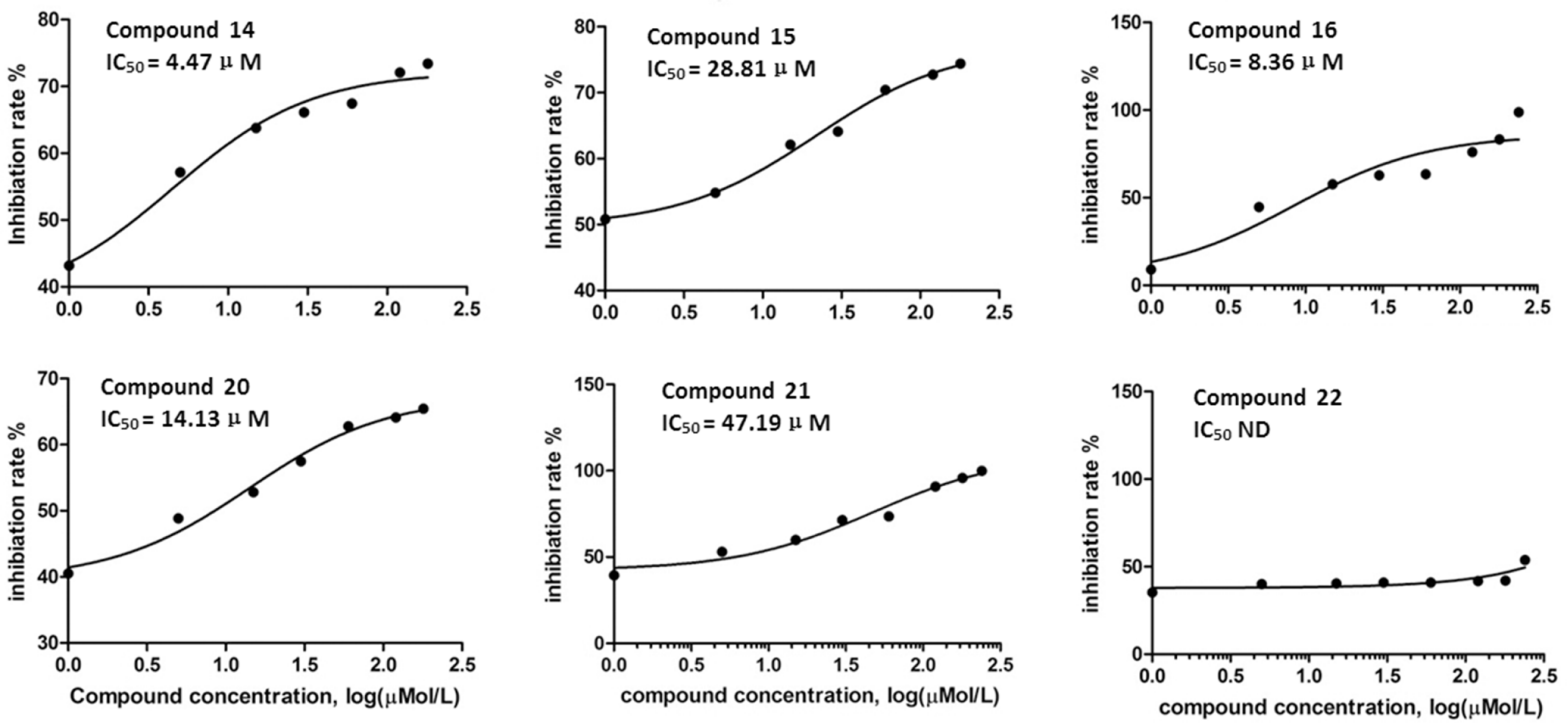
The inhibitory effect of the compounds on heparanase activity

Figure 1 shows the inhibitory effect of six compounds on HPA enzymatic activity; the six inhibitors, from strongest to weakest, were Nos. 14, 16, 20, 15, 21, and 22. These compounds were considered HPA inhibitors in this study.

### The effects of HPA inhibitors on cell growth

**Figure 2:**
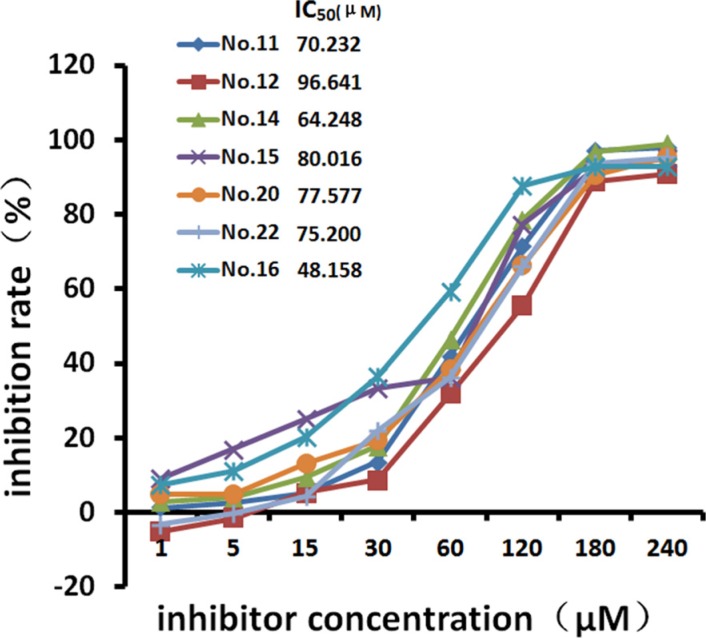
Growth inhibition curves for HeLa cells After 48 hours of treatment with different concentrations of the inhibitors, cell growth inhibition efficiency increased in a dose-dependent manner.

**Figure 3:**
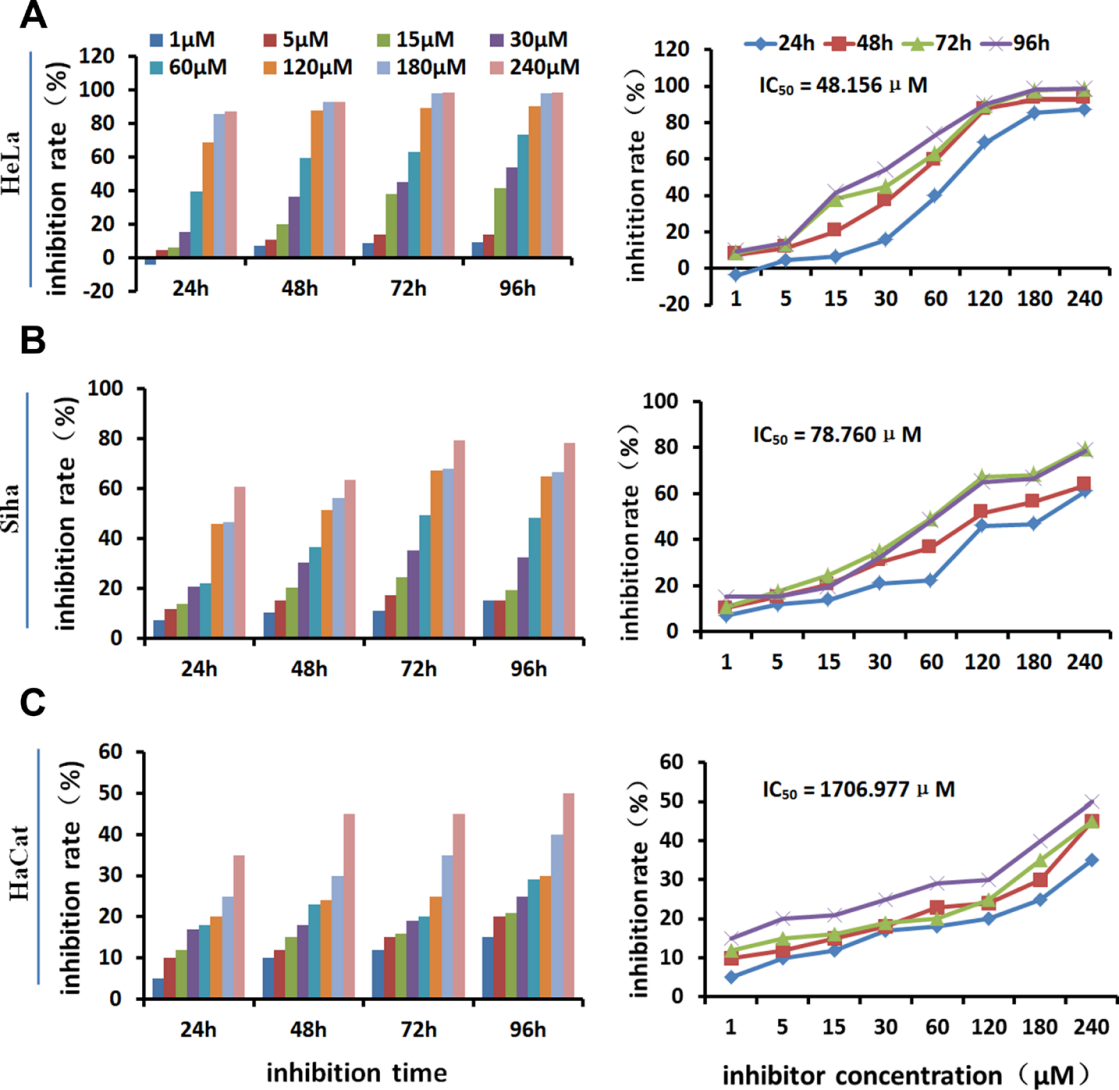
Growth-inhibiting effect of inhibitor No. 16 on different cell lines No. 16 inhibited the growth of HeLa (**A**) and Siha (**B**) cells in a time- and dose-dependent manner. It only slightly inhibited HaCaT cell growth (**C**).

MTT assays showed that the seven tested inhibitors dose-dependently inhibited the growth of HeLa cells after treatment for 48 hours (Table 1, Figure 2). Cell growth was inhibited by more than 30% by the 30 μM and 60 μM concentrations, and growth inhibition reached above 95% at the 240 μM concentration. The 48-hour IC_50_ values were 70.232 μM (No. 11), 96.641 μM (No. 12), 64.248 μM (No. 14), 80.016 μM (No. 15), 48.156 μM (No. 16), 77.577 μM (No. 20), and 75.200 μM (No. 22). Among them, No.16 showed the strongest inhibitory effect on cell growth as well as strong inhibition of HPA enzyme activity. It was therefore used to investigate the effects of HPA inhibition in different cell lines. Figure 3 shows that No. 16 suppressed the growth of HeLa and Siha cells, and displayed strong time and dose effects. HeLa growth was inhibited more than Siha growth (IC_50_ = 78.760 μM); No. 16 only slightly inhibited HaCat growth (IC_50_ = 1706.977 μM).

**Table 1:** Growth inhibition rates in HeLa cells treated with different concentrations of HPA inhibitors for 48 hs $\left({\bar{x}}_{\backslash *\text{MERGEFORMAT}}\pm \text{s},\%\right)$ and IC_50_ values

Concentration (μM)	No. 11	No. 12	No. 14	No. 15	No. 16	No. 20	No. 22
0	0	0	0	0	0	0	0
1	1.2237 ± 0.0789	−5.1478 ± 0.0626	2.9213 ± 0.1548	9.1202 ± 0.1662	7.4113 ± 0.9763	5.0057 ± 0.2468	−3.1278 ± 0.7049
5	2.6514 ± 0.0739	−1.5402 ± 0.4718	4.0529 ± 0.8879	17.0219 ± 0.7315	11.0361 ± 0.4665	5.0158 ± 0.0834	−0.3389 ± 0.0726
15	5.2631 ± 0.2936	5.3907 ± 0.3297	9.5748 ± 0.7606	25.1781 ± 0.9469	20.1973 ± 0.7792	13.2626 ± 0.3203	4.2667 ± 0.7289
30	13.6048 ± 1.2208	8.7551 ± 1.2071	17.9213 ± 1.1992	33.2189 ± 2.3399	36.5303 ± 0.4713	19.3996 ± 1.3401	22.0701 ± 0.9763
60	41.7965 ± 1.1885	31.7234 ± 1.2087	46.3622 ± 1.2154	36.1881 ± 0.5924	59.3469 ± 1.0994	38.4893 ± 0.2545	36.1334 ± 0.2850
120	71.3803 ± 1.1523	55.5327 ± 0.6710	78.5827 ± 0.5024	77.4678 ± 3.8195	87.6428 ± 0.8909	66.2954 ± 1.1366	66.0367 ± 0.8985
180	97.1849 ± 0.5248	89.0093 ± 0.4914	96.9764 ± 0.8598	92.4070 ± 3.0770	92.8868 ± 0.5366	90.5486 ± 1.1225	93.8527 ± 1.6700
240	98.0205 ± 0.4645	90.8935 ± 0.4160	98.9606 ± 0.5175	95.1657 ± 3.1108	92.9830 ± 0.2366	95.4357 ± 0.5249	95.1778 ± 0.3898
IC_50_ (μM)	70.232	96.641	64.248	80.016	48.158	77.577	75.200
F	17656.931	18287.542	15001.405	1406.687	15160.022	13954.993	13817.331
P	0.000	0.000	0.000	0.000	0.000	0.000	0.000

### The influence of inhibitors on cell morphology

**Figure 4:**
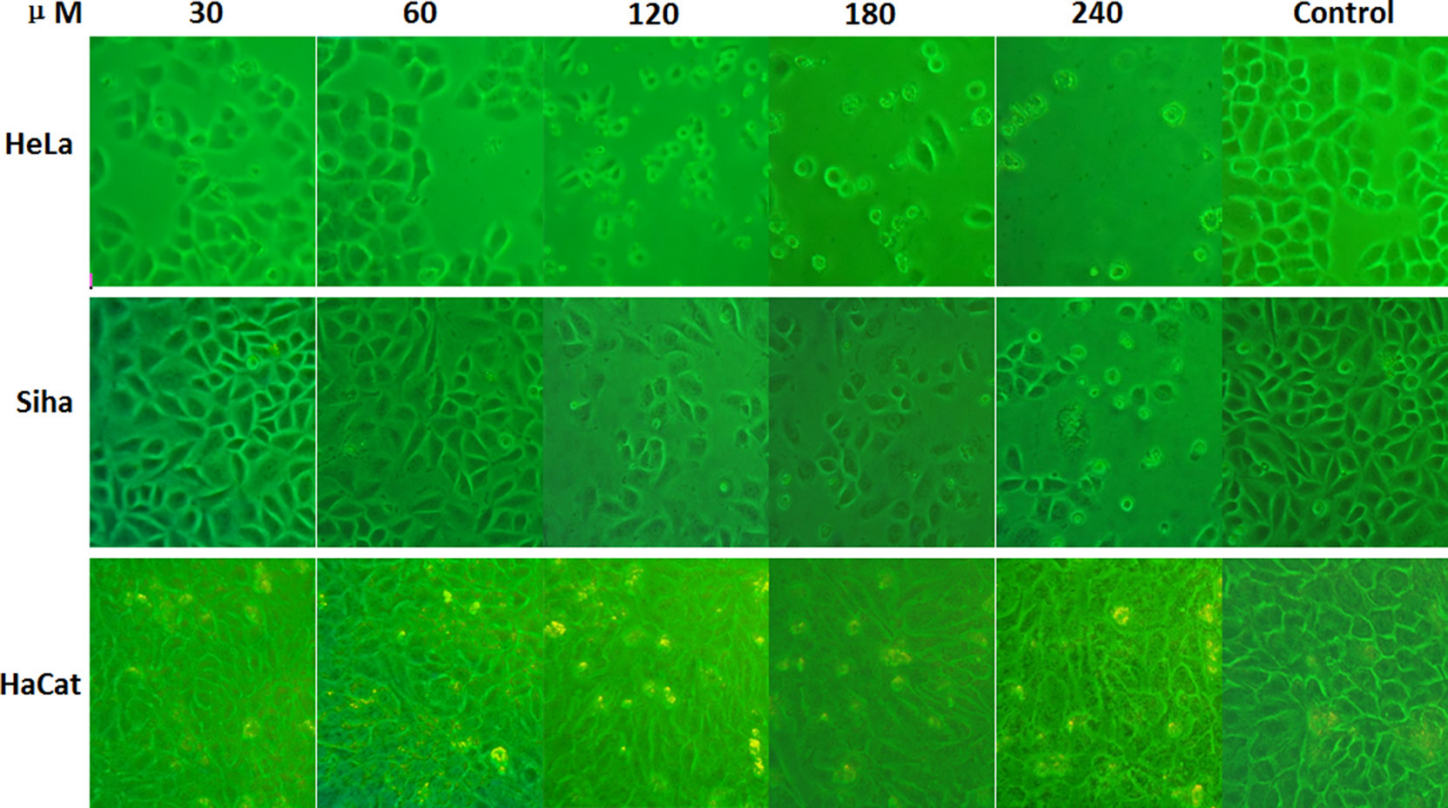
Effects of inhibitor No. 16 on cell growth morphology After 48 hours of treatment with different concentrations of No. 16, the morphology of HeLa and Siha cells as viewed under an inverted microscope at 200× magnification changed in a manner indicative of poor cell growth, including shielding, floating, round morphologies, and even death. Morphological changes were more obvious when the drug concentration was increased. Morphologic changes were more severe in HeLa than in Siha cells, and HaCaT cells were only mildly affected.

Cell morphology changed significantly after treatment with different concentrations of No. 16 (Figure 4). Treatment with 30 μM No. 16 for 48 hours inhibited growth. The cells did not spread well, and some cells began to float and turn round with intracellular granules. This phenomenon was more obvious at the 60 μM concentration. After 12 hours of 120 μM or 240 μM No. 16, cells could not adhere well to the well walls. They began to float and fall off after 24 hours and mostly died and shielded after 48 hours. Siha cells were impacted less than HeLa cells by No. 16, and the impact on HaCaT cells was minimal.

### No. 16 sensitized cells to the cytotoxic effect of nedaplatin

**Figure 5:**
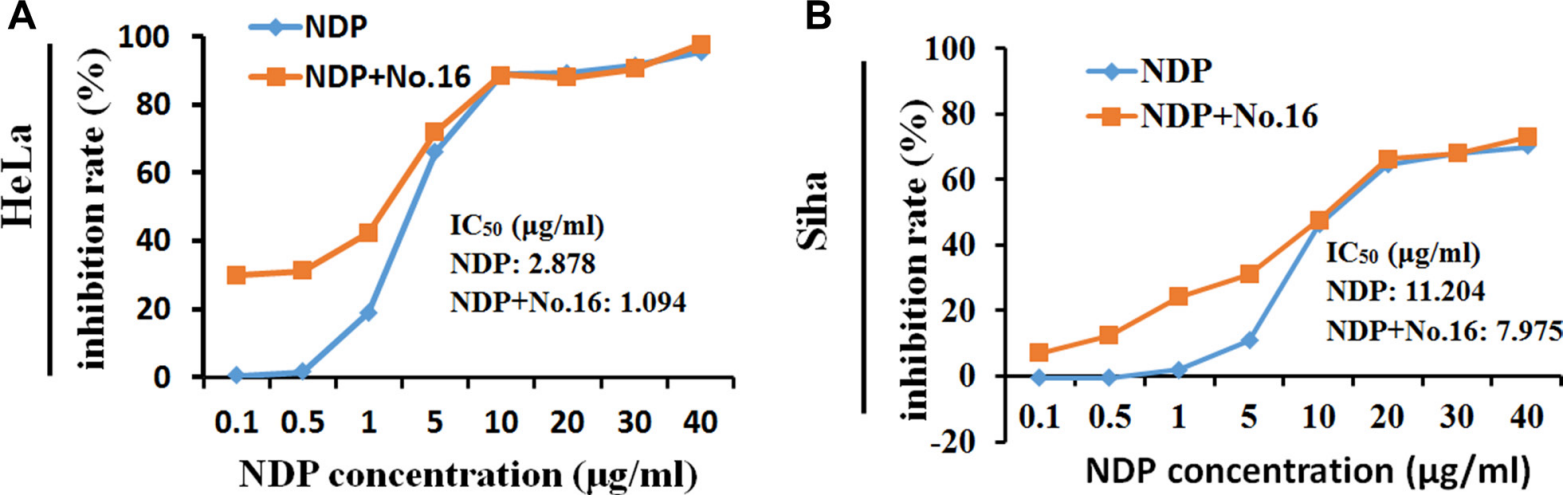
No. 16 inhibitor sensitized cell response to nedaplatin After 48 hours of treatment with 10 μM No. 16 inhibitor and different concentrations of nedaplatin, cytotoxicity in response to low concentrations of nedaplatin increased in HeLa (**A**), and Siha (**B**) cells.

The MTT assay showed that the growth of HeLa (Figure 5A) and Siha (Figure 5B) cells was inhibited by 48 hours of treatment with nedaplatin alone (IC_50_ = 3.396 μg/ml and 11.204 μg/ml, respectively). After combined treatment with 10 μM No.16 and nedaplatin for 48 hours, growth inhibition IC_50_ values for nedaplatin in HeLa (1.094 μg/ml) and Siha (7.975 μg/ml) cells decreased. Combined treatment with No.16 inhibitor increased the sensitivity of these cells to low concentrations of nedaplatin.

### Inhibiting effect on cell migration

**Figure 6:**
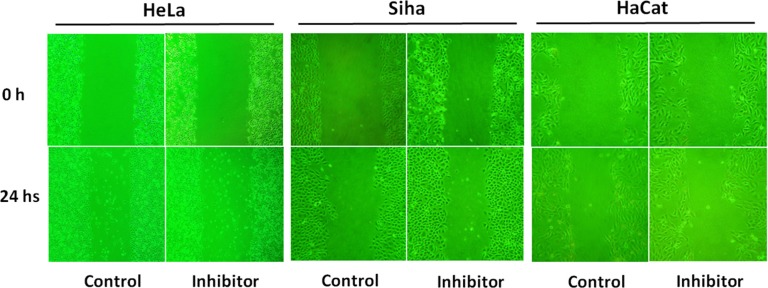
The effect of inhibitor No. 16 on cell migration ability After 24 hours of treatment with 50 μM of inhibitor No. 16, the migration ability of HeLa and Siha cells as viewed under an inverted microscope at 200× magnification was reduced; HaCaT cells were only mildly affected.

As shown in Figure 6, the scratch width narrowed due to cell migration. Compared to control groups, 50 μM No. 16 inhibited the migration of both HeLa and Siha cells, and inhibitory potency was stronger in HeLa cells. The impact on HaCaT cells was minimal.

### The influence of No. 16 on cell cycle progression and apoptosis

**Figure 7:**
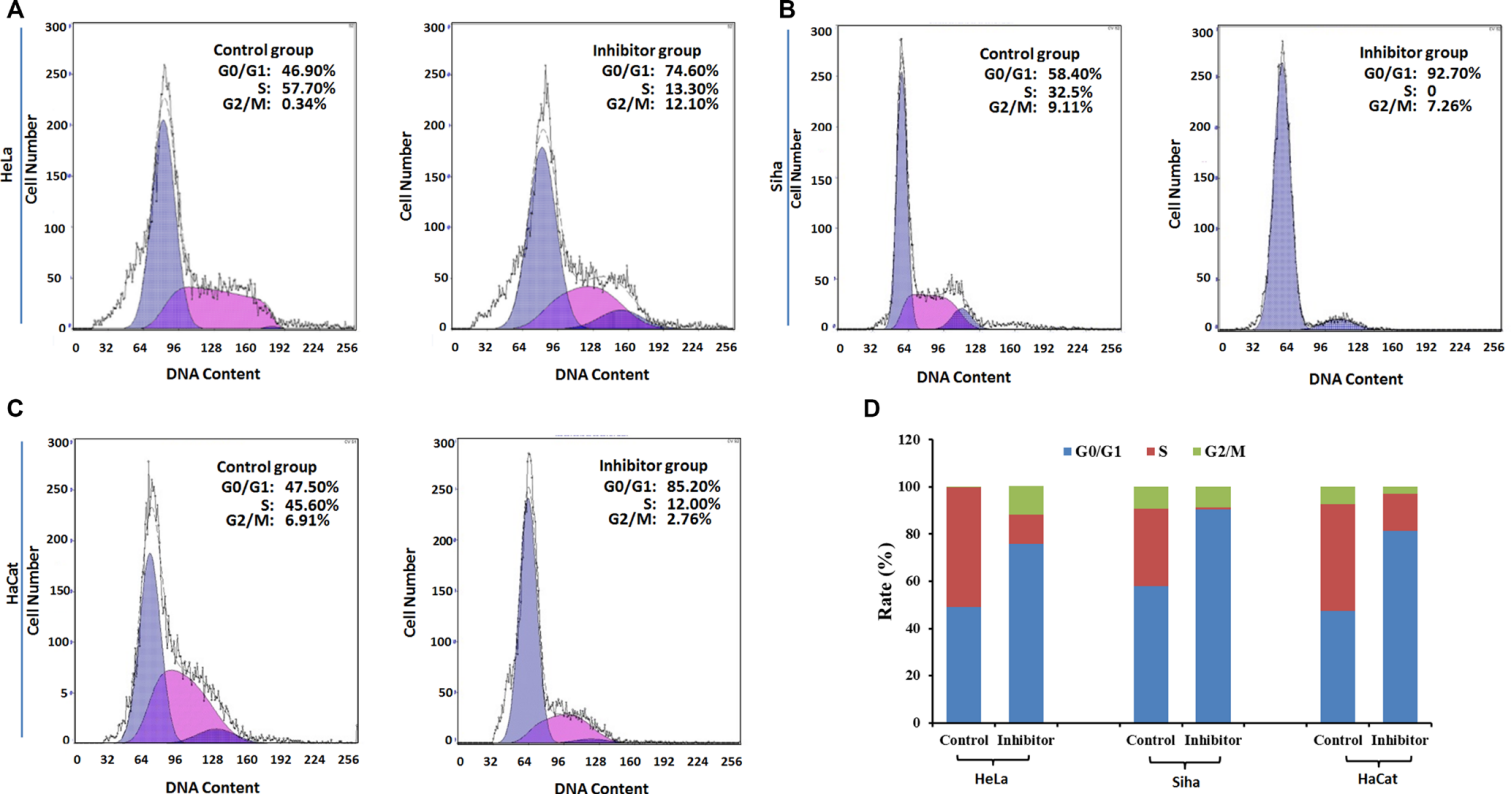
The effect of inhibitor No. 16 on cell cycle progression in HeLa, Siha, and HaCaT cells (**A**), (**B**) and (**C**) Graphs showing cell cycles. Compared with control cells, the ratio of G_0_ phase cells increased in inhibitor groups, *p* = 0.0004 for HeLa, *p <* 0.0001 for Siha, *p* = 0.0001 for HaCaT (**D**). In contrast, the ratio of S phase cells decreased in inhibitor groups, *p* = 0.0012 for HeLa, *p* = 0.0004 for Siha, *p* = 0.001 for HaCaT.

**Figure 8:**
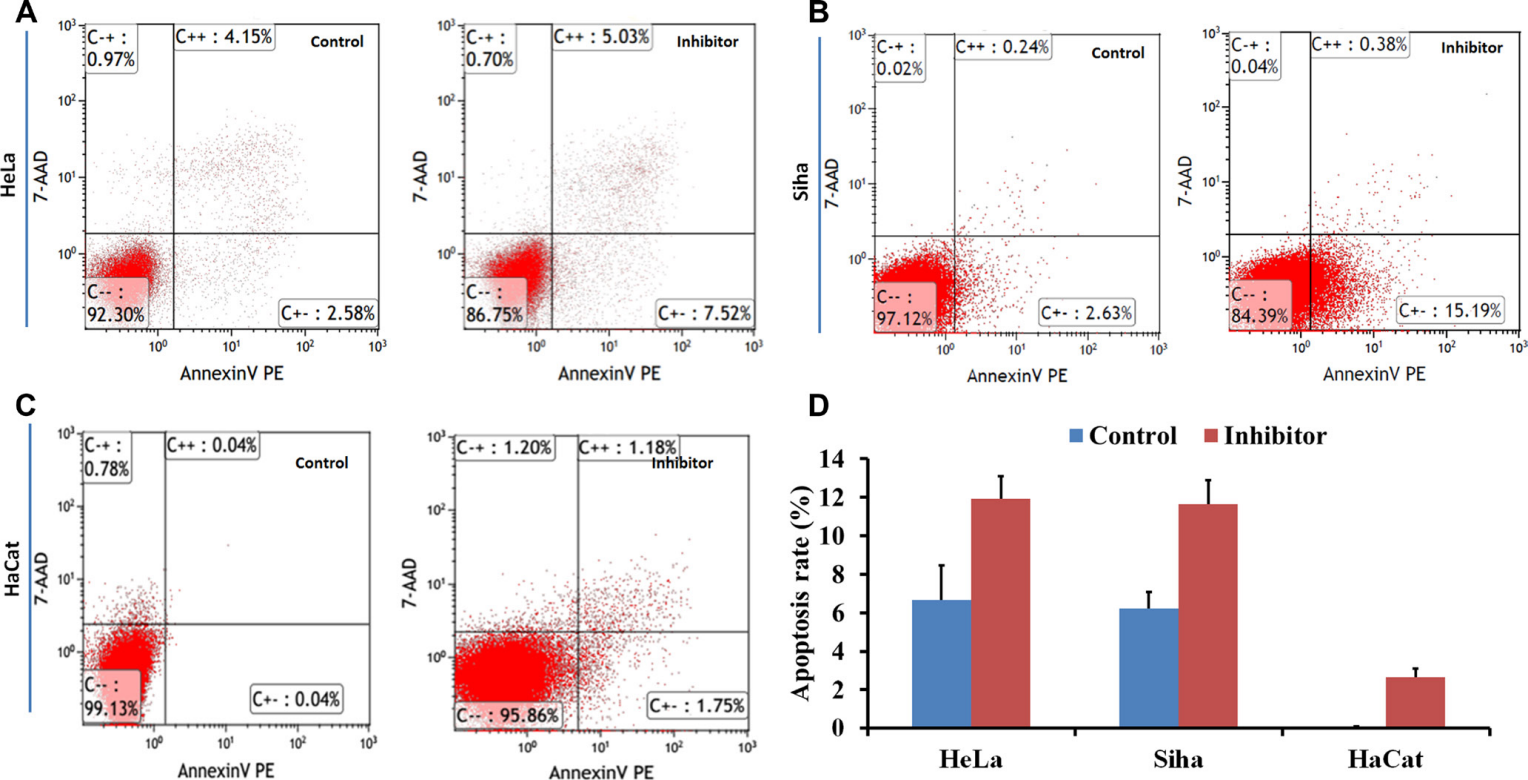
The effect of inhibitor No. 16 on cell apoptosis in HeLa, Siha, and HaCaT cells Flow cytometry was performed to determine cell apoptosis. After 48 hours of treatment with 50 μM No. 16, cell apoptosis rates of HeLa (**A**) and Siha (**B**) were higher than in control groups, all *p <* 0.05; (**D**) similar effects were observed in HaCaT cells (**C**).

Flow cytometry showed that G_0_/G_1_ phase arrest increased, and the S phase population decreased, in HeLa, Siha, and HaCaT cells (Figure 7); cell apoptosis also increased following treatment with 50 μM No. 16 for 48 hours (Figure 8).

### The influence of No 16 on HPA, p53, c-Myc, and h-TERT expression

**Figure 9:**
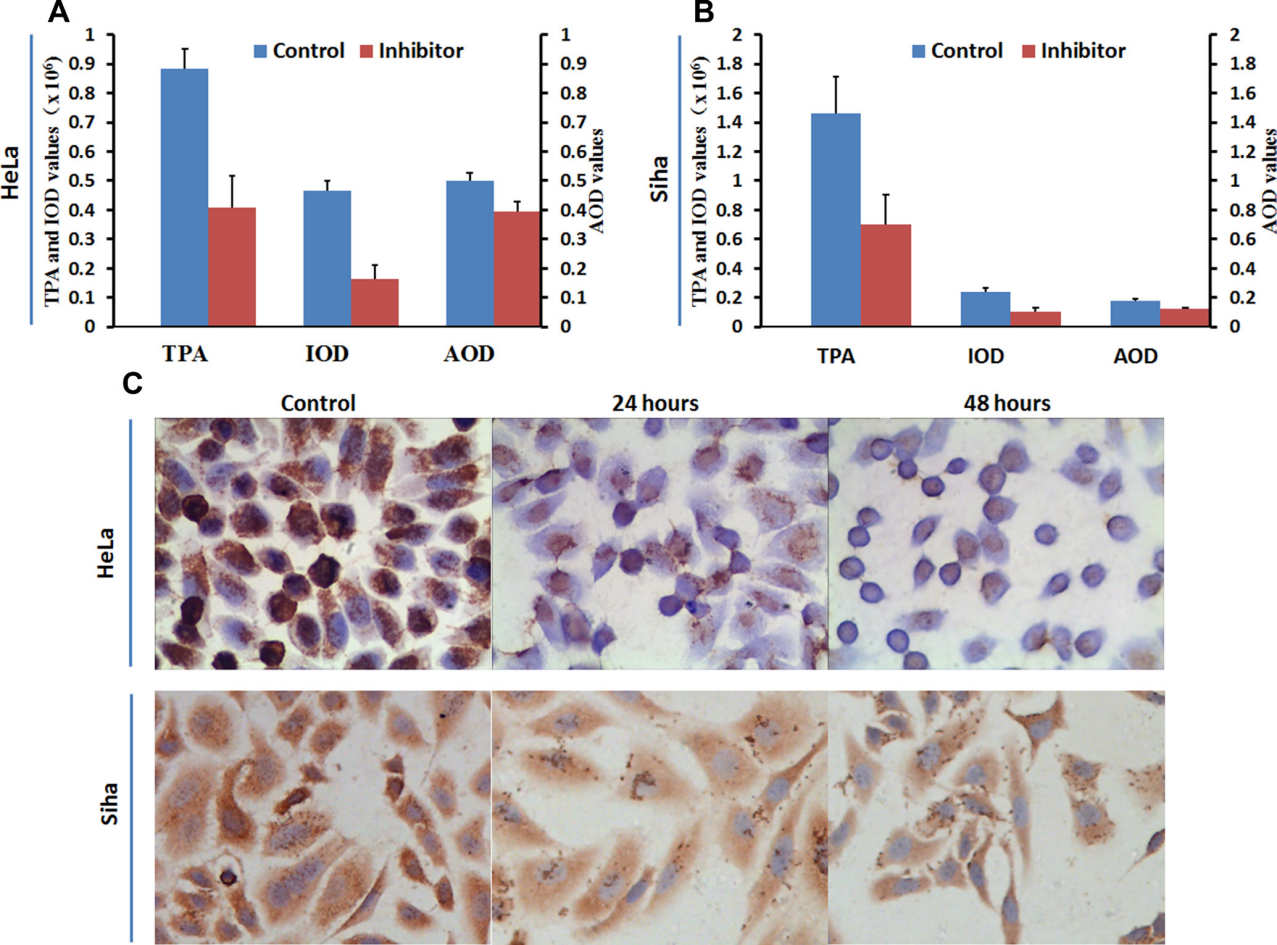
Influence of inhibitor No. 16 on HPA protein levels in HeLa and Siha cells Mean optical density, total cumulative positive area, and integrated optical density values for HPA immunocytochemical staining in HeLa (**A**) and Siha (**B**) cells treated with No. 16 for 48 hours were lower than those in control cells, all *p* values < 0.01. HPA levels in HeLa cells were higher than in Siha cells. Coarse brown-colored HPA particles were located in HeLa cell nuclei and cytoplasm and in Siha cell cytoplasm with a diffuse distribution (**C**). HPA protein levels were reduced in HeLa nuclei and cytoplasm and in Siha cytoplasm after 24 hours of treatment. HPA staining was further reduced in HeLa nuclei, but no further change in HPA levels was observed in Siha cells, after 48 hours of treatment. All pictures were taken at 400× magnification. TPA: total positive area; IOD: integral optical density; AOD: average optical density

**Figure 10:**
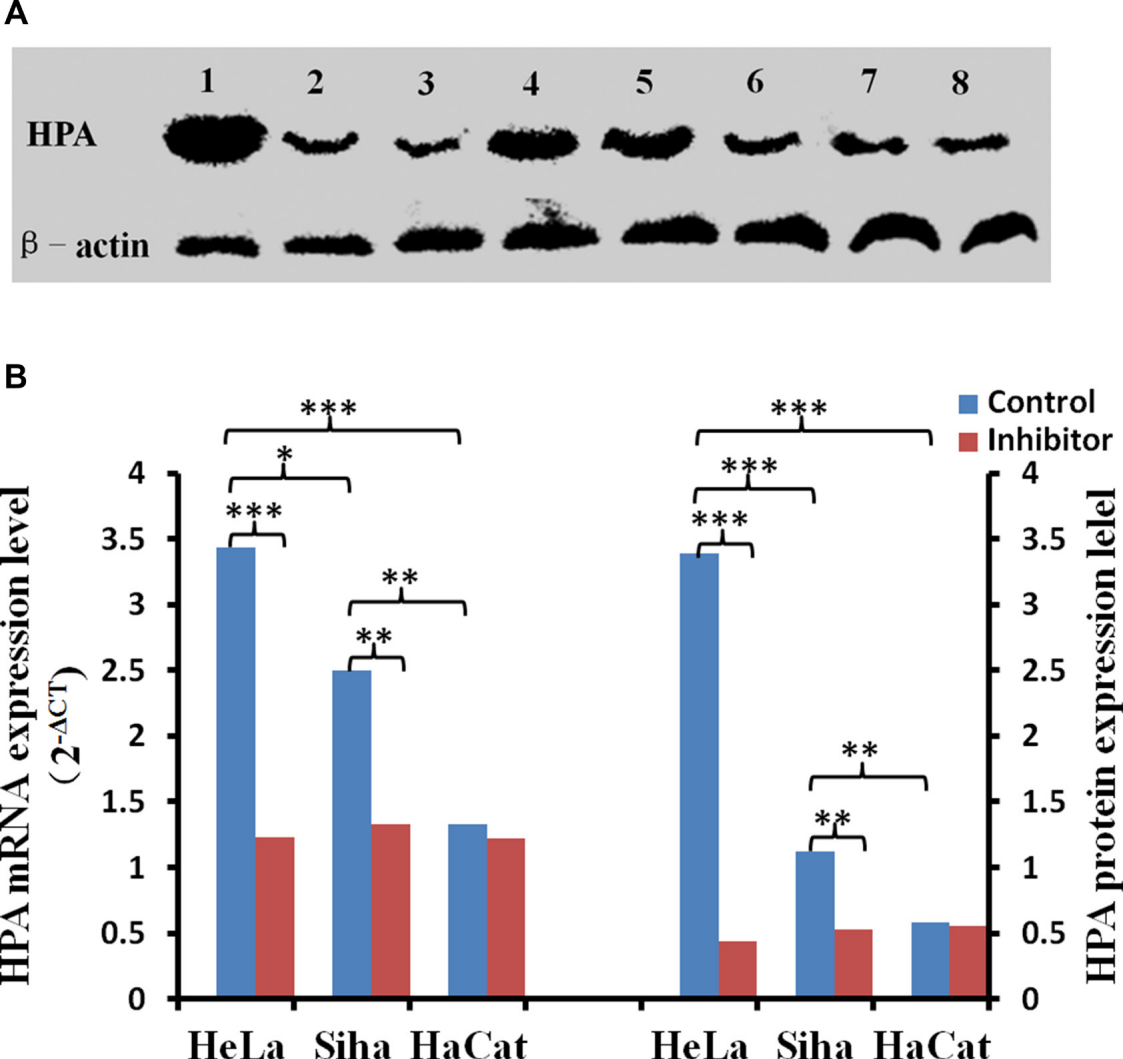
The effect of inhibitor No. 16 on HPA mRNA and protein levels in cervical cancer cells After treatment with 50 μM No. 16, HPA mRNA and protein levels decreased in HeLa and Siha cells. (**A**) Lanes 1, 2, and 3 correspond to HPA immunoblots for HeLa cells after 0 h, 24 h, and 48 h of inhibitor treatment, respectively; lanes 4, 5, and 6 show the same for Siha cells. Lanes 8 and 9 are HPA immunoblots in HaCat cells after 0 h and 48 h of inhibitor treatment. (**B**) Statistical results for relative levels of HPA mRNA and protein in cervical cancer and HaCaT cells treated with 50 μM No. 16 for 48 h. *p < 0.05, **p < 0.01, ***p < 0.001.

**Figure 11:**
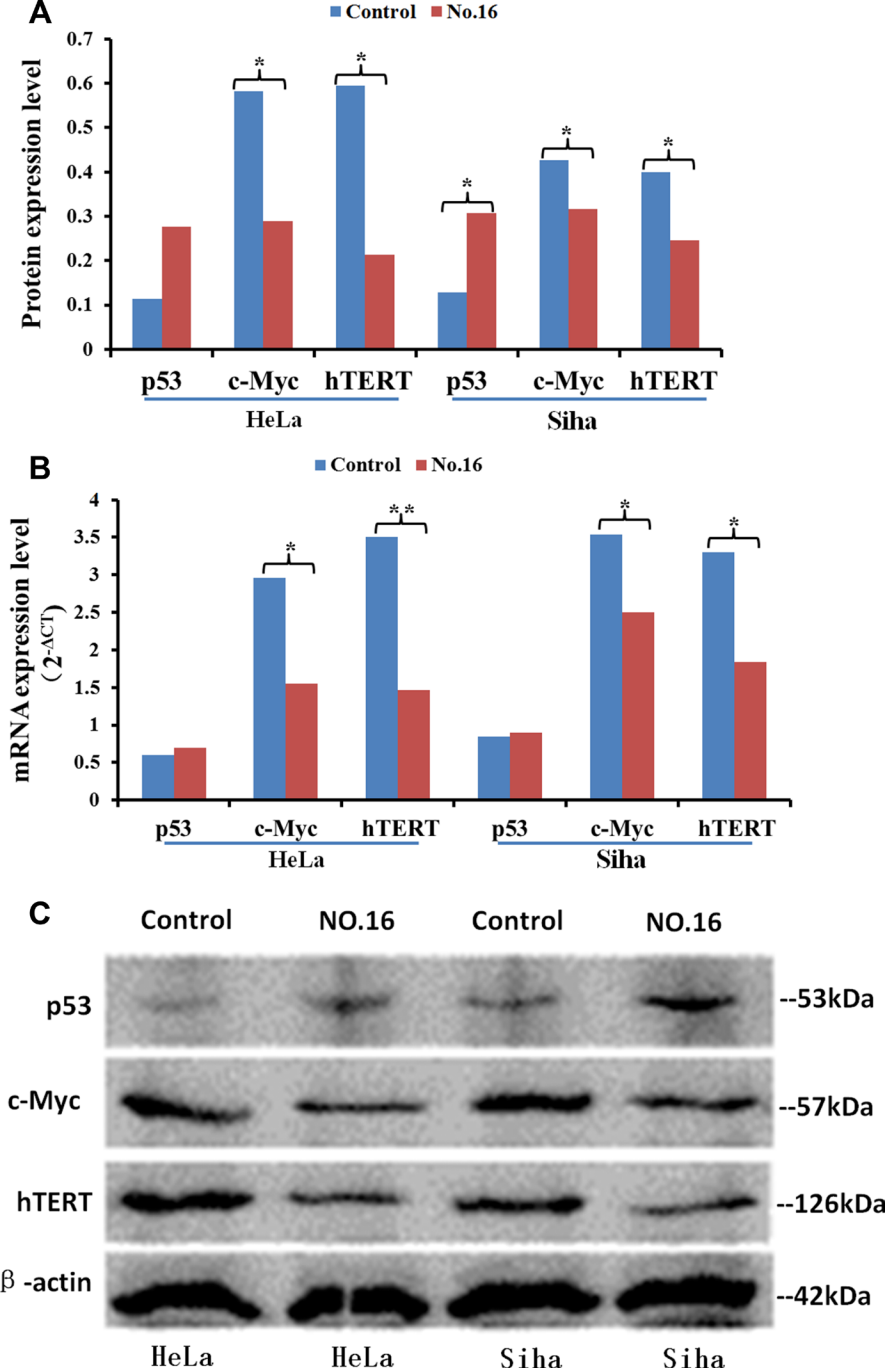
The effect of inhibitor No. 16 on p53, c-Myc, and hTERT levels in cervical cancer cells . After treatment with 50 μM No. 16, c-myc and h-TERT protein (**A**, **C**) and mRNA (**B**) levels decreased in HeLa and Siha cells. p53 protein, but not mRNA, levels also increased. *p < 0.05, **p < 0.01.

No. 16 suppressed the expression of HPA, and this down-regulation was stronger in HeLa cells than in Siha cells. Immunocytochemical staining showed that HPA expression was abundant in untreated HeLa and Siha cells (Figure 9). HPA protein was located in both the cytoplasm and nucleus of HeLa cells, but only in the cytoplasm of Siha cells. HPA levels were higher in HeLa cells than in Siha cells. 50 μM No. 16 reduced HPA levels after 24 hours of treatment, and this reduction was stronger after 48 hours. TPA, IOD and AOP values differed between the inhibitor-treated groups and control groups according to the analysis conducted with immunohistochemical quantitative software. The results of western blot and real time PCR confirmed that No. 16 dose-dependently reduced HPA protein and mRNA levels (Figure 10). Western blot and PCR also showed that 48 hours of 50 μM No. 16 increased p53 expression and decreased h-TERT and c-Myc expression in both HeLa and Siha cells (Figure 11).

## DISCUSSION

Although screening and early treatment and the use of the HPV vaccine have reduced its incidence worldwide, many women are still diagnosed with advanced, recurrent, or persistent cervical cancer. These patients do not respond well to any currently available treatments. Recently, a phase III clinical trial (GOG204) in the United States found that targeted drugs can improve the survival of patients with advanced, recurrent, or persistent cervical cancer [5]. The development of new targeted treatments may therefore help improve therapy for this disease. Here, we successfully synthesized 13 new compounds that have inhibitory effects on cervical cancer-associated enzyme activity. These inhibitors also inhibited cell growth by increasing apoptosis and reduced HPA expression, suggesting that they may be beneficial for cervical cancer therapy.

The core of the DMBO molecule contains a homologous pyran ring on which carbon atoms are replaced by nitrogen, resulting in a 1,3-O, N, hexaheterocyclic molecule. 13-O, N heterocyclic compounds have attracted increasing attention because of their potential roles in cancer. Recently, a growing number of heterocyclic compounds have been developed to inhibit HPA enzyme activity; examples include benzothiazole, benzoxazole, benzimidazole derivatives, and the imidazole pyrimidine class [15–18]. Compared to DMBO, the spiroheterocyclic HPA inhibitors we developed here have stronger inhibitory activity and increase cell apoptosis by antagonizing HPA. By increasing cell apoptosis, these compounds inhibit the growth of HeLa and Siha cells and downregulate HPA expression *in vitro*, especially in HeLa cells with high baseline HPA expression.

Single cisplatin chemotherapy has been considered the most effective treatment for advanced and recurrent cervical cancer, with a response rate of 38% [1]. Carboplatin, nedaplatin, and other platinum drugs used as second-line chemotherapy are also beneficial for recurrent cervical cancer patients who fail to respond to first-line cisplatin treatment [19]. Increased HPA expression can promote resistance of cancer cells to chemotherapy, and drug resistance can be overcome by inhibiting HPA activity [20]. Therefore, we examined the effects of combined nedaplatin and No. 16 inhibitor treatment on cervical cancer cells. A low concentration of the HPA inhibitor enhanced the cytotoxicity of nedaplatin, which is very important for the treatment of cisplatin-resistant cervical cancer.

The persistent HPV infection is a main cause of cervical cancer and precancerous lesions, and the relationship between HPA and HPV also has attracted increasing attention. By attaching predominantly to heparan sulfate chains on the cell surface and basement membrane, HPV virus particles enter host cells after a series of protein conformational changes. Inhibiting HPA activity dramatically reduces the release of the HPV16 virus from the extracellular matrix, as well as cellular uptake and infection. Conversely, exogenous HPA can activate this process [21]. HPV E6 and E7 proteins, two main carcinogenic proteins, can cause cellular transformation via their actions on multiple signal transduction pathways [22]. By increasing p53 ubiquitination, HPV E6 reduces the ability of p53 to activate the promotor of HPA, leading to a higher HPA expression [23, 24]. E6 stabilizes h-TERT mRNA and increases h-TERT expression, thus leading to an increase in telomerase activity [22]. Tang *et al*. [25] found that h-TERT upregulates heparanase expression by binding to c-Myc and recruiting this complex to the heparanase promoter in gastric cancers. Our study showed that HPA inhibitors can simultaneously upregulate p53 expression and inhibit the expression of HPA, h-TERT, and c-Myc. Therefore, we hypothesize that HPA inhibitors may reduce cellular proliferation and HPA expression in cervical cancer by restoring p53 activity and downregulating the expression of h-TERT and c-Myc. All of the evidence mentioned above indicates that HPA participates in HPV infection and the development of cervical cancer through various mechanisms. Inhibiting HPA function may therefore be particularly effective in preventing and treating cervical cancer.

In summary, combined cytotoxic drugs and molecular target agent therapy is a promising new method of cervical cancer treatment. The No. 16 inhibitor we developed here inhibited both HPA enzyme activity and the growth and migration of HPV18/16-positive cervical cancer cell lines. Additionally, this inhibitor increased the cytotoxicity of nedaplatin in HeLa and Siha cells while only slightly affecting HPV-negative HaCaT human normal keratinocytes. Our study suggests that the targeted inhibition of HPA may prove to be an effective cervical cancer therapy and that it is worthy of further study.

## MATERIALS AND METHODS

### Synthesis of new HPA inhibitors

We synthesized two series of 13 compounds by changing the type and position of substituents on the benzene ring of DMBO. The 1,3-O, N-Spiroheterocyclic derivatives of DMBO were expected to be HPA inhibitors. Their molecular structures were characterized using IR, NMR, MS, and other methods. Characteristics of all 13 compounds are listed in Table 2.

**Table 2:** Summary of heparanase inhibitor synthesis

Product number	Chemical formula	Molecular mass	Structure of the product	Melting point (°C)	Product number	Chemical formula	Molecular mass	Structure of the product	Melting point (°C)
10	C_21_H_24_N_2_O_3_	352.42686	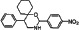	108.2–109.4	17	C_22_H_26_NO_2_Cl	371.90034	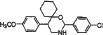	91.2–92.1
11	C_21_H_24_NOCl	341.87436	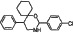	127.7–128.5	18	C_22_H_26_NO_2_F	355.44674	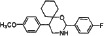	101.4–102.6
12	C_21_H_24_NOF	325.42076	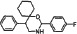	95.2–93.3	19	C_22_H_26_NO_2_Br	416.35134	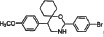	86.1–87.2
13	C_21_H_24_NOBr	386.32536	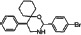	150.3–152.1	20	C_23_H_29_NO_2_	351.48186	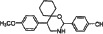	105.4–106.7
14	C_21_H_23_NOF_2_	343.41222	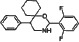	130.4–131.2	21*	C_22_H_25_NO_2_F_2_	373.4382	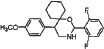	131.7–132.6
15	C_22_H_26_N_2_O_4_	382.45284	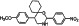	111.3–112.5	22	C_22_H_25_NO_2_F_2_	373.4382	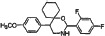	128.7–129.6
16	C_22_H_26_NO_2_F	355.44674	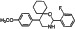	171.3–172.5					

* DMBO.

### Cell lines and reagents

HPV-18-positive HeLa and HPV-16-positive Siha cervical cancer cell lines and HaCaT human normal keratinocytes were obtained from the Medical Institute of Experimental Animals of the Chinese Academy of Medical Sciences (Shanghai, China) and from Beijing Dingguo Biotechnology (Beijing, China). The heparan degrading enzyme assay kit was purchased from Takara Inc. (Cat. #MK412, Tokyo, Japan). RPMI-1640 (#SH30023.01B), MEM (#SH30024.01B), and H-DMEM (#SH30022.01B) medium supplemented with 100 U/ml penicillin and streptomycin and 0.25% Trypsin (#SC30042.01B) was purchased from Hyclone Laboratories Inc. (Logan, UT). Fetal bovine serum (FBS) without phage and endotoxin was from HangZhou Sijiqing Company (Hangzhou, China). Dimethyl Sulfoxide (DMSO) and Thiazolyl Blue (MTT) were from Sigma-Aldrich (Saint Louis, MO) and Genview Scientific Inc. (El Monte, CA), respectively. The Cycletest Plus DNA Reagent Kit and Annexin V Apoptosis Detection Kit I were obtained from BD Biosciences (#BD559763, San Jose, CA). Rabbit anti-HPA1 antibody (#SC-25825) was obtained from Santa Cruz Biotechnology Inc. (Dallas, TX). The immunohistochemical detection system, consisting of ready-to-use PV-9001 and a DAB chromogenic reagent kit, was purchased from Zhongshan Jinqiao Company (Beijing, China). Secondary goat anti-rabbit antibody conjugated with horseradish peroxidase was from bioWORLD (#21310018–1, Dublin, OH). Anti-P53 (#AB1101), anti-TERT (#AB183105), anti-c-Myc (#AB32072), and anti-β-actin (#AB8226) antibodies were purchased from Abcam (Cambridge, MA). TRIzol^®^ Plus RNA Purification Kit (#12183–555) and TaqMan^®^ Gene Expression Assays for HPA (#4351372), HERT (#4331182), c-Myc (#4331182), TP53 (#4331182), and human TATA box binding protein (#4331182) were purchased from Ambion (Carlsbad, CA).

### Detection of HPA activity inhibition

According to the instructions of the Heparan Degrading Enzyme Assay Kit, different concentrations (1, 5, 15, 30, 60, 120, 180, and 240 μM) of inhibitors were added into the reaction system containing 50 μl biological heparin and 31 mIU human recombinant HPA. The final reaction volume of each vial was 100 μl. The reaction vials were incubated at 37°C for 45 minutes. The reaction products were moved to 96-well plates and incubated at 37°C for 45 minutes. After washing the plate three times, anti-biotin conjugated with horseradish peroxidase and 100 μl horseradish peroxidase substrate was added to each well and incubated at 37°C for 30 minutes. Then, 100 μl of terminating liquid was added into each well. OD values were examined at a wavelength of 450 nm. Each concentration was tested in three separate wells. GraphPad Prism 5 (GraphPad Prism Software Inc., San Diego) was used to calculate the inhibitory IC_50_ values.

### Cell culture and inhibitor treatment

Cells were cultured in improved medium supplemented with 10% FBS and kept at 37°C in a 38% humidified atmosphere containing 5% CO_2_. Logarithmic phase cells were used for the experiment and were treated with different concentrations of different medications for different amounts of time. The blank groups consisted of only improved medium alone without cells or drug compounds, and the normal control groups were single-cell suspensions without inhibitors or nedaplatin. Inhibitors were made into 10 mM stock solutions with DMSO, filtered, and kept at room temperature away from light. These inhibitor stock solutions were diluted to the required concentration (1, 5, 15, 30, 60, 120, 180, or 240 μM) before every experiment. The final doses of nedaplatin were 0.1, 0.5, 1, 5, 10, 20, 30, or 40 mg. In order to determine whether an HPA inhibitor sensitized cells to nedaplatin, 10 μM of inhibitor number 16 (No. 16) was added into each experimental well with nedaplatin.

### MTT assay

200 μl of single-cell suspension at a density of 5 × 10^4^ cells/ml was seeded into 96-well plates. When cells were adherent, the medium was replaced with 200 μl of medium containing different concentrations of inhibitor or nedaplatin, and the cells were further incubated for 24 hours to 96 hours respectively. 20 μl MTT solution (5 mg/L in phosphate buffered saline (PBS)) was added to each well 4 hours before the treatment ended. The cells were then incubated for another 4 hours, after which the supernatants were removed. The cells were totally solubilized in 150 μl DMSO. OD values were measured at the wavelength of 492 nm with a reference wavelength of 630 nm. Inhibition rate (IR) in the cells was calculated as follows: IR = ((OD2-OD0)-(OD1-OD0))/(OD2-OD0) × 100%, where OD0 was the OD value of blank wells, OD2 was the value of normal control wells, and OD1 was the value of experimental wells. Each condition was replicated in six separate wells.

### Cell morphology observation

Single-cell suspensions at a density of 5 × 10^4^ cells/ml were seeded into 6-well plates. When cells were adherent, the medium was replaced with different concentrations of No. 16 inhibitor. Cell morphology changes were observed with an inverted microscope after incubation for 24, 48, or 72 hours. The inhibitor was left out of the control wells.

### Cell wound healing assay

Straight lines 0.5 cm apart from each other were marked on the bottoms of culture dishes opposite the label lines. Logarithmic phase cells were seeded into 35 mm culture dishes. Nicks were made 0.5 cm apart on the bottom of the dish along the labeled lines with a 200 μl sterile pipette tip after cells were close to saturated. Dishes were then washed with sterile PBS three times, 2% FBS medium containing 0 or 50 μM of No. 16 inhibitor was added, and dishes were incubated for 24 hours. The scratch width was photographed under phase contrast on an inverted microscope with a digital camera (100× magnification). The experiment was repeated three times.

### Flow cytometry

2 ml of single-cell suspensions at a density of 5 × 10^4^/ml were seeded into 6-well plates. When cells were adherent, the medium was replaced with 50 μM No.16 inhibitor or an equal volume of medium without inhibitor (as a control). Each condition was replicated in six separate wells. After incubation for 48 hours, the cells were washed with precooled PBS twice, and collected into centrifuge tubes for centrifugation (800 rps, 5 min). Flow cytometry was used to detect cell cycle stage and apoptosis in labeled cells according to the manufacturer's instructions. The experiment was repeated three times.

### Western blotting

Cells were washed with precooled PBS after being treated with inhibitor for 48 hours, and cell lysis solution was added to extract protein. The sample concentration was measured by the bicinchoninic acid method. 30 μg of protein was separated by 10% polyacrylamide gel electrophoresis (PAGE) and transferred to polyvinylidene difluoride membranes. After blocking in non-fat milk solution at room temperature, the membranes were incubated with diluted primary antibodies against either proteins of interest or β-actin overnight at 4°C. After consecutive washes, the membranes were incubated with secondary antibody (diluted 1:5000) at room temperature for 1 h. Then, the membranes were developed with enhanced chemiluminescence and imaged with ImageQuant LAS4000 (GE Healthcare Life Sciences, Pittsburgh, PA). The grey values of protein bands were analyzed using Image J (National Institutes of Health, USA). The relative levels of proteins of interest were calculated in comparison to the internal reference β-actin. The experiment was repeated three times.

### Immunocytochemical staining

Single-cell suspensions at a density of 5 × 10^4^ cells/ml were seeded into the bottom of 24-well plates with coverslips. When the cells were adherent, different concentrations of inhibitor were added to the wells. The coverslips were removed after 24, 48, or 96 hours of incubation, and cells were fixed with 4% paprformaldehyde at room temperature. The S-P immunocytochemical method was used to test the expression of HPA according to the instructions. After incubation with primary HPA antibody (Santa Cruz, diluted at 1:100) overnight at 4°C, slides were colored with DAB and counterstained with hematoxylin. HPA-positive HeLa slides were used as positive controls. Negative control slides were incubated with PBS instead of primary antibody. Three separate wells were processed for each group. The experiment was repeated twice.

Brown immunocytochemistry-labeled HPA granules were located in the cytoplasm and nucleus. Five areas in each slide were randomly chosen and photographed at 400× magnification. Image Pro Plus software (Media Cybernetics, Warrendale, PA) was used to analyze and measure the total positive area (TPA), integrated optical density (IOD), and average optical density (AOD).

### Real-time PCR

Levels of HPA, p53, c-Myc, and hTERT mRNA were analyzed by real-time PCR. mRNA was extracted from cells and separated and purified with Trizol. The total RNA concentration was measured with an ultraviolet spectrophotometer. The quality of total RNA was assessed using 1.5% agarose gel electrophoresis. The synthesis of cDNA and the preparation of PCR amplification system were carried out in accordance with kit instructions. The reaction volume was 20 μl, and PCR was performed using the following settings: 50°C for 2 min, 95°C for 10 min, and 40 reaction cycles of 95°C for 15 s and 60°C for 1 min using a type 7300 real-time PCR system (Applied Biosystems company, USA). The endogenous human TBP gene was used as an internal control. Results were expressed in relative amounts as described elsewhere [15]. Three separate PCR amplification reactions were run for each condition.

### Statistical analysis

The SPSS 17.0 software package (SPSS Inc., Chicago, IL) was used. Statistical evaluation of data was carried out using student's *t*-test for two groups and ANOVA for comparisons between more than two groups. *p <* 0.05 was considered statistically significant.

## References

[1] Thigpen T, Shingleton H, Homesley H, Lagasse LBlessing J (1981). Cis-platinum in treatment of advanced or recurrent squamous cell carcinoma of the cervix: a phase II study of the Gynecologic Oncology Group. Cancer.

[2] Moore DH, Blessing JA, McQuellon RP, Thaler HT, Cella D, Benda J, Miller DS, Olt G, King S, Boggess JFRocereto TF (2004). Phase III study of cisplatin with or without paclitaxel in stage IVB, recurrent, or persistent squamous cell carcinoma of the cervix: a gynecologic oncology group study. Journal of clinical oncology.

[3] Eskander RNTewari KS (2014). Beyond angiogenesis blockade: targeted therapy for advanced cervical cancer. Journal of gynecologic oncology.

[4] Seol HJ, Ulak R, Ki KDLee JM (2014). Cytotoxic and targeted systemic therapy in advanced and recurrent cervical cancer: experience from clinical trials. The Tohoku journal of experimental medicine.

[5] Tewari KS, Sill MW, Long HJ, Penson RT, Huang H, Ramondetta LM, Landrum LM, Oaknin A, Reid TJ, Leitao MM, Michael HEMonk BJ (2014). Improved survival with bevacizumab in advanced cervical cancer. The New England journal of medicine.

[6] Fux L, Ilan N, Sanderson RDVlodavsky I (2009). Heparanase: busy at the cell surface. Trends in biochemical sciences.

[7] Pisano C, Vlodavsky I, Ilan NZunino F (2014). The potential of heparanase as a therapeutic target in cancer. Biochemical pharmacology.

[8] McKenzie EA (2007). Heparanase: a target for drug discovery in cancer and inflammation. British journal of pharmacology.

[9] Shinyo Y, Kodama J, Hongo A, Yoshinouchi MHiramatsu Y (2003). Heparanase expression is an independent prognostic factor in patients with invasive cervical cancer. Annals of oncology.

[10] Zeng C, Ke ZF, Luo WR, Yao YH, Hu XR, Jie W, Yin JBSun SJ (2013). Heparanase overexpression participates in tumor growth of cervical cancer *in vitro* and *in vivo*. Medical oncology.

[11] Hasengaowa, Kodama J, Kusumoto T, Shinyo Y, Seki N, Nakamura K, Hongo AHiramatsu Y (2005). Loss of basement membrane heparan sulfate expression is associated with tumor progression in endometrial cancer. European journal of gynaecological oncology.

[12] Kodama J, Shinyo Y, Hasengaowa, Kusumoto T, Seki N, Nakamura K, Hongo AHiramatsu Y (2005). Loss of basement membrane heparan sulfate expression is associated with pelvic lymph node metastasis in invasive cervical cancer. Oncology reports.

[13] Dafni H, Cohen B, Ziv K, Israely T, Goldshmidt O, Nevo N, Harmelin A, Vlodavsky INeeman M (2005). The role of heparanase in lymph node metastatic dissemination: dynamic contrast-enhanced MRI of Eb lymphoma in mice. Neoplasia.

[14] Basappa, Murugan S, Kavitha CV, Purushothaman A, Nevin KG, Sugahara KRangappa KS (2010). A small oxazine compound as an anti-tumor agent: a novel pyranoside mimetic that binds to VEGF, HB-EGF, and TNF-alpha. Cancer letters.

[15] Courtney SM, Hay PA, Buck RT, Colville CS, Phillips DJ, Scopes DI, Pollard FC, Page MJ, Bennett JM, Hircock ML, McKenzie EA, Bhaman M, Felix R (2005). Furanyl-1,3-thiazol-2-yl and benzoxazol-5-yl acetic acid derivatives: novel classes of heparanase inhibitor. Bioorganic & medicinal chemistry letters.

[16] Courtney SM, Hay PA, Buck RT, Colville CS, Porter DW, Scopes DI, Pollard FC, Page MJ, Bennett JM, Hircock ML, McKenzie EA, Stubberfield CRTurner PR (2004). 2,3-Dihydro-1,3-dioxo-1H-isoindole-5-carboxylic acid derivatives: a novel class of small molecule heparanase inhibitors. Bioorganic & medicinal chemistry letters.

[17] Xu YJ, Miao HQ, Pan W, Navarro EC, Tonra JR, Mitelman S, Camara MM, Deevi DS, Kiselyov AS, Kussie P, Wong WCLiu H (2006). N-(4-{[4-(1H-Benzoimidazol-2-yl)-arylamino]-methyl}-phenyl)-benzamide derivatives as small molecule heparanase inhibitors. Bioorganic & medicinal chemistry letters.

[18] Pan W, Miao HQ, Xu YJ, Navarro EC, Tonra JR, Corcoran E, Lahiji A, Kussie P, Kiselyov AS, Wong WCLiu H (2006). 1-[4-(1H-Benzoimidazol-2-yl)-phenyl]-3-[4-(1H-benzoimidazol-2-yl)-phenyl]-urea derivatives as small molecule heparanase inhibitors. Bioorganic & medicinal chemistry letters.

[19] Takekuma M, Kuji S, Tanaka A, Takahashi N, Abe MHirashima Y (2015). Platinum sensitivity and non-cross-resistance of cisplatin analogue with cisplatin in recurrent cervical cancer. Journal of gynecologic oncology.

[20] Ramani VC, Zhan F, He J, Barbieri P, Noseda A, Tricot GSanderson RD (2016). Targeting heparanase overcomes chemoresistance and diminishes relapse in myeloma. Oncotarget.

[21] Surviladze Z, Sterkand RTOzbun MA (2015). Interaction of human papillomavirus type 16 particles with heparan sulfate and syndecan-1 molecules in the keratinocyte extracellular matrix plays an active role in infection. The Journal of general virology.

[22] Chen J (2015). Signaling pathways in HPV-associated cancers and therapeutic implications. Reviews in medical virology.

[23] Hirshoren N, Bulvik R, Neuman T, Rubinstein AM, Meirovitz AElkin M (2014). Induction of heparanase by HPV E6 oncogene in head and neck squamous cell carcinoma. J Cell Mol Med.

[24] Baraz L, Haupt Y, Elkin M, Peretz TVlodavsky I (2006). Tumor suppressor p53 regulates heparanase gene expression. Oncogene.

[25] Tang B, Xie R, Qin Y, Xiao YF, Yong X, Zheng L, Dong HYang SM (2016). Human telomerase reverse transcriptase (hTERT) promotes gastric cancer invasion through cooperating with c-Myc to upregulate heparanase expression. Oncotarget.

[26] Schmittgen TDLivak KJ (2008). Analyzing real-time PCR data by the comparative C(T) method. Nature protocols.

